# Statistics of Insanity

**Published:** 1854-01-01

**Authors:** Alexander Morrison

**Affiliations:** PHYSICIAN TO THE SURREY COUNTY LUNATIC ASYLUM, ETC.


### STATISTICS OF INSANITY.

BY SIR ALEXANDER MORRISON, M.D., PHYSICIAN TO THE SURREY COUNTY LUNATIC ASYLUM, ETC.

In a Paper lately laid before the Society for Improving the Condition of the Insane, established in 1842, by the *late* Earl of Shaftesbury, the following highly gratifying statement is given by Sir Alexander Morrison, of the result of treatment in the Public Establishments of which he has been Physician during the last thirty years:—

| | | |
|---|---|---|
| The admissions amounted to ... | 6779 | |
| | | 6779 |
| Of whom were removed by friends or others during treatment ... | 454 | |
| Remaining in the establishments | 1587 | |
| Leaving to be accounted for ... | 4738 | |
| | | 6779 |

| | | |
|---|---|---|
| Of whom were discharged ... | Uncured ... | 806 |
| | Died... | 1440 |
| | Recovered ... | 2492 |

Of the 806 uncured, 173 were either paralytic, epileptic, or idiotic.

The causes of the disorder assigned in 1428 of the cases recovered were as under:—

| | |
|---|---|
| Hereditary predisposition existed in ... | 386 |
| Intemperance... | 181 |
| Pregnancy, child-bearing, abortion, lactation, &c... | 172 |
| Disappointments, reverses, embarrassments, losses, or privations ... | 116 |
| Religious excitement ... | 76 |
| Grief ... | 62 |
| Disappointed affection ... | 50 |
| Anxiety, vexation ... | 43 |
| Terror... | 39 |
| Blows on the head, falls ... | 23 |
| Epilepsy... | 23 |
| Paralysis ... | 8 |
| Causes of more rare occurrence | 219 |

Of the remaining cases for which no cause was assigned, the disorder had previously occurred in 270.

The causes of death in 1431 cases were as under:—

| | |
|---|---|
| Paralysis (in general the cause of the disorder intemperance) ... | 284 |
| Exhaustion (chiefly after great cerebral excitement) ... | 196 |
| Pulmonary consumption ... | 164 |
| Epilepsy... | 135 |
| Diarrhœa ... | 126 |
| Apoplexy ... | 100 |
| Decay of nature—old age... | 52 |
| Convulsions ... | 29 |
| Diseased lungs ... | 29 |
| Fever ... | 27 |
| Hydrothorax ... | 25 |
| Abscesses and ulcers... | 25 |
| Cerebral disease... | 20 |
| Erysipelas ... | 18 |
| Disease of the heart or great vessels ... | 18 |
| Asiatic cholera ... | 18 |
| General debility... | 16 |
| Dysentery, ulcerated intestines... | 13 |
| Suicide ... | 13 |
| Tabes, marasmus, atrophy... | 11 |
| Cancer ... | 10 |
| Bronchitis ... | 10 |
| Asphyxia ... | 9 |

| | | | |
|---|---|---|---|
| Dropsy | 8 | Diseased Stomach | 2 |
| Pneumonia | 7 | Lumbar abscess | 2 |
| Asthma | 7 | Hæmorrhoidal discharge | 2 |
| Enteritis | 6 | Chorea | 2 |
| Gangrene | 6 | Ruptured liver | 1 |
| Wounds, falls | 5 | Ruptured spleen | 1 |
| Cynanche | 5 | Hernia | 1 |
| Pleuritis | 4 | Hemiplegia | 1 |
| Diseased liver, bilious cholera | 4 | Delirium tremens | 1 |
| Diseased bladder, cystitis, ischuriæ | 4 | Concussion | 1 |
| Diseased ovaria | 4 | Spinal disease | 1 |
| Peritonitis | 3 | Scrofula | 1 |
| Influenza | 3 | Burn | 1 |

---

